# Primary total elbow arthroplasty with ulna fixation for a Jupiter type IID posterior Monteggia fracture-dislocation: a case report

**DOI:** 10.1016/j.xrrt.2026.100897

**Published:** 2026-10-05

**Authors:** Takanobu Higashi, Shunsuke Takahara, Hiroyuki Takayama, Natsuko Nakagawa

**Affiliations:** Department of Orthopaedic Surgery, Hyogo Prefectural Kakogawa Medical Center, Kakogawa, Hyogo, Japan

**Keywords:** Posterior Monteggia fracture-dislocation, Total elbow arthroplasty, Open reduction and internal fixation, Elderly, Osteoporosis, Coronoid fracture

## Introduction

Type IID Monteggia fracture-dislocation is a subtype of Bado type II, defined by Jupiter et al in 1991 as a complex ulna fracture extending from the olecranon to the diaphysis.^1^, ^3^, ^4^, ^8^ Among the Bado type II subtypes, the IID subtype is the most complex and surgically demanding.^3^ Despite advances in surgical techniques and improved understanding of the injury, post-operative outcomes of the IID subtype remain suboptimal compared with those of other subtypes.^3^, ^6^ Recently, a coronoid-centric classification system (the Mayo classification) has been proposed for complex elbow fracture-dislocations in which the dorsal cortex of the ulna is fractured. This classification divides these injuries into 3 patterns according to whether the fractured coronoid process remains attached to the ulnar metaphysis (trans-olecranon fracture-dislocations), the olecranon (Monteggia-variant fracture-dislocations), or neither (trans-ulnar basal coronoid fracture-dislocations).^2^, ^13^ Among these patterns, the trans-ulnar basal coronoid type is the most difficult to manage and carries the least favorable prognosis.^13^ Clinical challenges include limited range of motion (ROM), increased risk of delayed union or nonunion, and poor functional scores.^4^, ^9^, ^13^ The main causes of these unfavorable results are considered to be the presence of concomitant coronoid fractures, avulsion fractures at ligament insertions, and radial head comminution.^9^, ^21^ Although timely anatomical reduction and stable fixation of the fragments are essential to minimize complications, persistent instability, and poor functional outcomes,^3^, ^6^, ^9^ achieving this is often difficult in elderly patients because of poor bone quality, fracture comminution, articular fragmentation, and existing comorbidities.^6^, ^7^, ^15^, ^21^

Primary total elbow arthroplasty (TEA) is increasingly utilized for managing unreconstructible distal humerus fractures in elderly individuals.^10^ Compared with open reduction and internal fixation (ORIF), TEA offers faster rehabilitation and better short-term outcomes for these injuries.^12^ Although TEA has been proposed as a treatment option for unreconstructible elbow fractures beyond the distal humerus,^25^ reports involving other fracture types are limited.^16^, ^20^

This case report describes an elderly patient with osteoporosis who sustained a fall resulting in a Jupiter type IID posterior Monteggia fracture-dislocation with severe comminution. Treatment using a combination of primary TEA and ORIF resulted in excellent clinical outcomes.

## Case presentation

### Patient characteristics

An 89-year-old woman sustained an injury to her left elbow following a fall from standing height. She was referred to our department 9 days post-injury with a diagnosis of a posterior Monteggia fracture-dislocation. On physical examination, swelling and tenderness were noted, but there were no open wounds. Neurovascular status was intact. Her medical history included osteoporosis (bone mineral density 59% of the young adult mean), diabetes mellitus (glycated hemoglobin, 8.2%), and mild dementia.

### Imaging

Radiographs and computed tomography revealed a posterior elbow dislocation, a comminuted proximal ulna fracture, and a radial head fracture consistent with a type IID Monteggia fracture-dislocation (Fig. 1). Both 2-dimensional and 3-dimensional computed tomography images showed a comminuted fracture of the proximal ulna extending into the diaphysis and involving the olecranon, coronoid, crista supinatoris, and sublime tubercle (Fig. 2, *A-E*), as well as a radial head fracture classified as Mason type IV.^11^ The coronoid fracture, classified as O'Driscoll type III subtype 2, consisted of multiple articular fragments (Fig. 2, *F*).^14^ By the coronoid-centric classification of proximal trans-ulnar fracture-dislocations (the Mayo classification), this injury represents a trans-ulnar basal coronoid fracture-dislocation, in which the coronoid is attached to neither the olecranon nor the ulnar metaphysis.^2^

**Figure 1:**
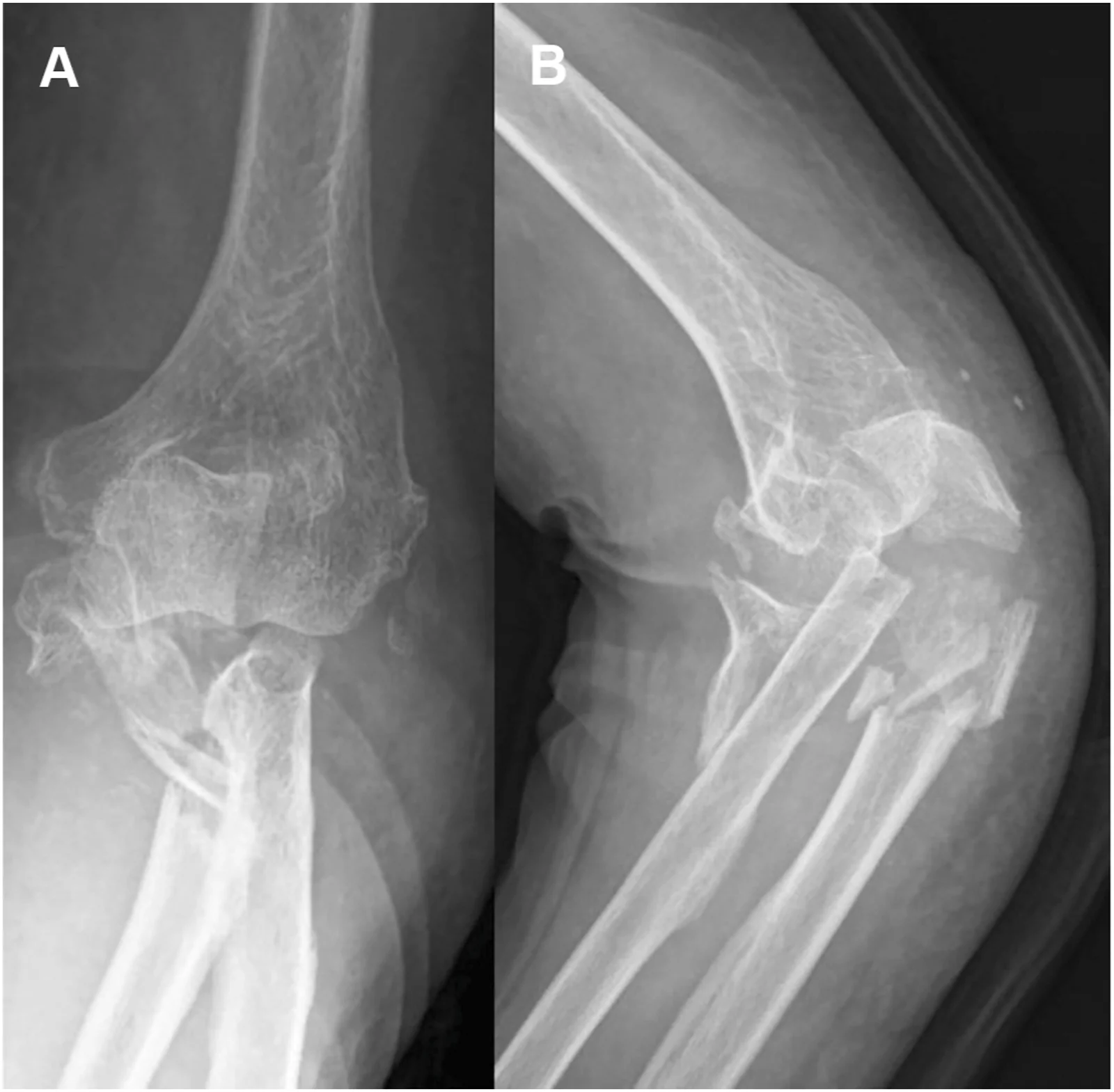
(**A** and **B**) Initial elbow radiographs demonstrating a posterior Monteggia fracture-dislocation with a comminuted proximal ulna fracture and an associated radial head fracture.

**Figure 2:**
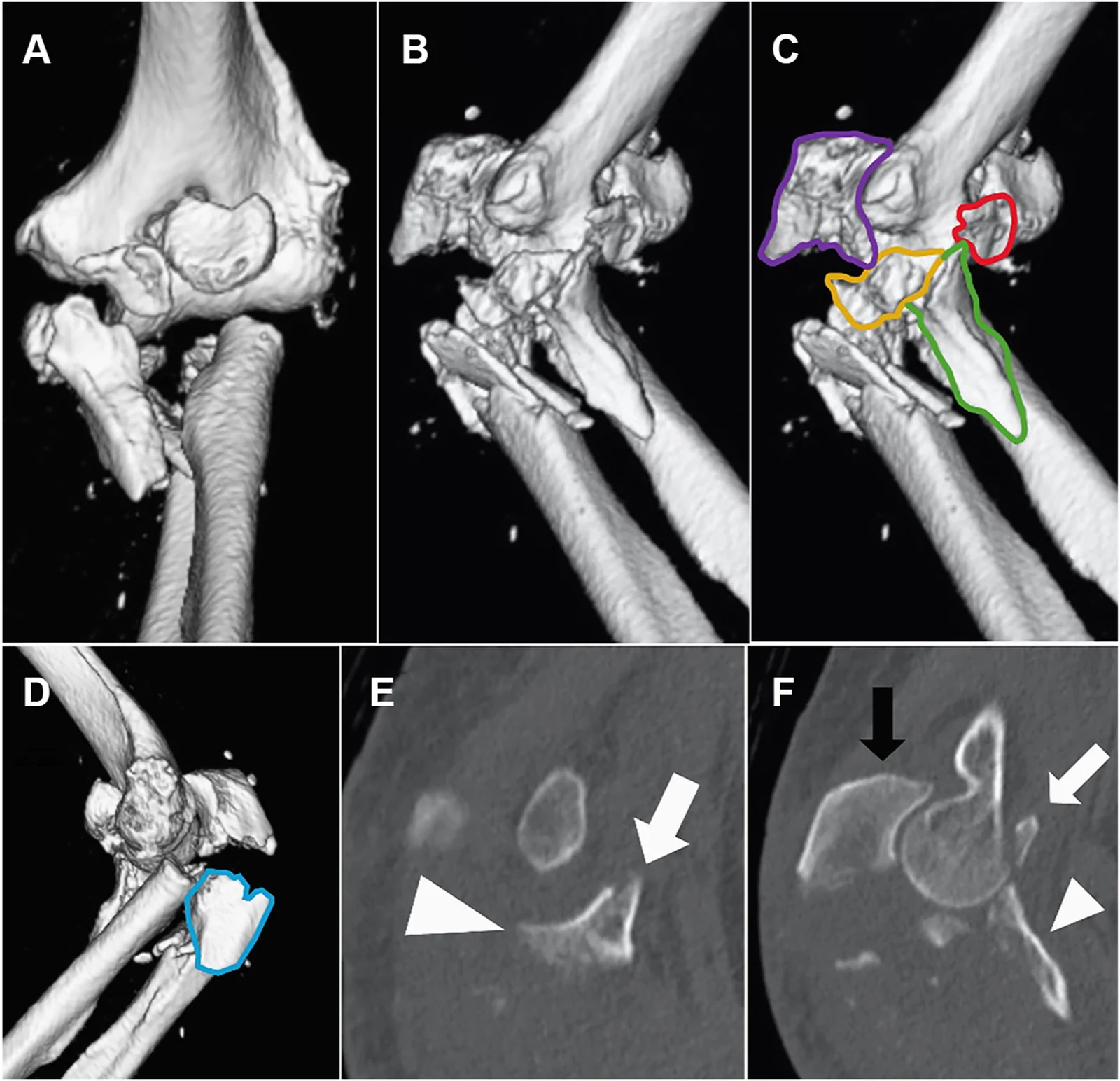
(**A–D**) Three-dimensional computed tomography (CT) of the elbow showing a Jupiter type IID fracture pattern with a Mason type IV radial head fracture. (**C**) Multifragmentary proximal ulna fracture involving the olecranon (purple *circle*), anterolateral facet of the coronoid (red *circle*), coronoid base (green *circle*), and sublime tubercle (yellow *circle*). (**D**) Posterolateral proximal ulna fragment including the crista supinatoris (blue *circle*). (**E**) Sagittal CT view depicting the olecranon fracture (black *arrow*), anterolateral facet fragment (white *arrow*), and coronoid base fragment (*arrowhead*). (**F**) The coronoid fracture, classified as O'Driscoll type III subtype 2, showed multiple fragments within the articular surface (white *arrow* and *arrowhead*).

### Surgical procedure

A primary TEA combined with ORIF was performed 12 days after the injury. The procedure was conducted under general anesthesia and a regional block, with the patient in the prone position.

A 20-cm posterior incision was made with the olecranon fragment used as a landmark. The ulnar nerve was identified, released, and protected without anterior transposition. The olecranon fragment was reflected proximally to expose the fracture site and the elbow joint. The following fragments were identified: a fragment including the anterolateral facet of the coronoid (red, Fig. 2, *C*), a fragment including the coronoid base (green, Fig. 2, *C*), a fragment including the sublime tubercle (yellow, Fig. 2, *C*), a posterolateral fragment including the crista supinatoris (blue, Fig. 2, *D*), and a radial head fragment. The anterolateral facet fragment and the radial head fragment were excised. The remaining fragments were mobilized, and the interposed fibrous granulation tissue was débrided.

Following the manufacturer's technique for the Nexel Total Elbow System (Zimmer Biomet, Warsaw, IN, USA), the distal humerus was resected with reference to the transepicondylar axis, and the humeral trial component was inserted. After resection of 1 cm of the radial neck, the coronoid base fragment, together with the sublime tubercle fragment and the crista supinatoris fragment, was temporarily fixed with Kirschner wires to temporarily reconstruct the proximal ulna. Using these fragments as landmarks for positioning the ulnar component, the depth and rotational alignment were determined, ensuring smooth flexion–extension, and the ulnar trial component was then inserted. With the trial component in place, the 3 fragments were fixed with 2 plates (VariAx Hand 2.3, Stryker, Kalamazoo, MI, USA) (Fig. 3). The residual coronoid tip was then excised to prevent impingement during flexion.

**Figure 3:**
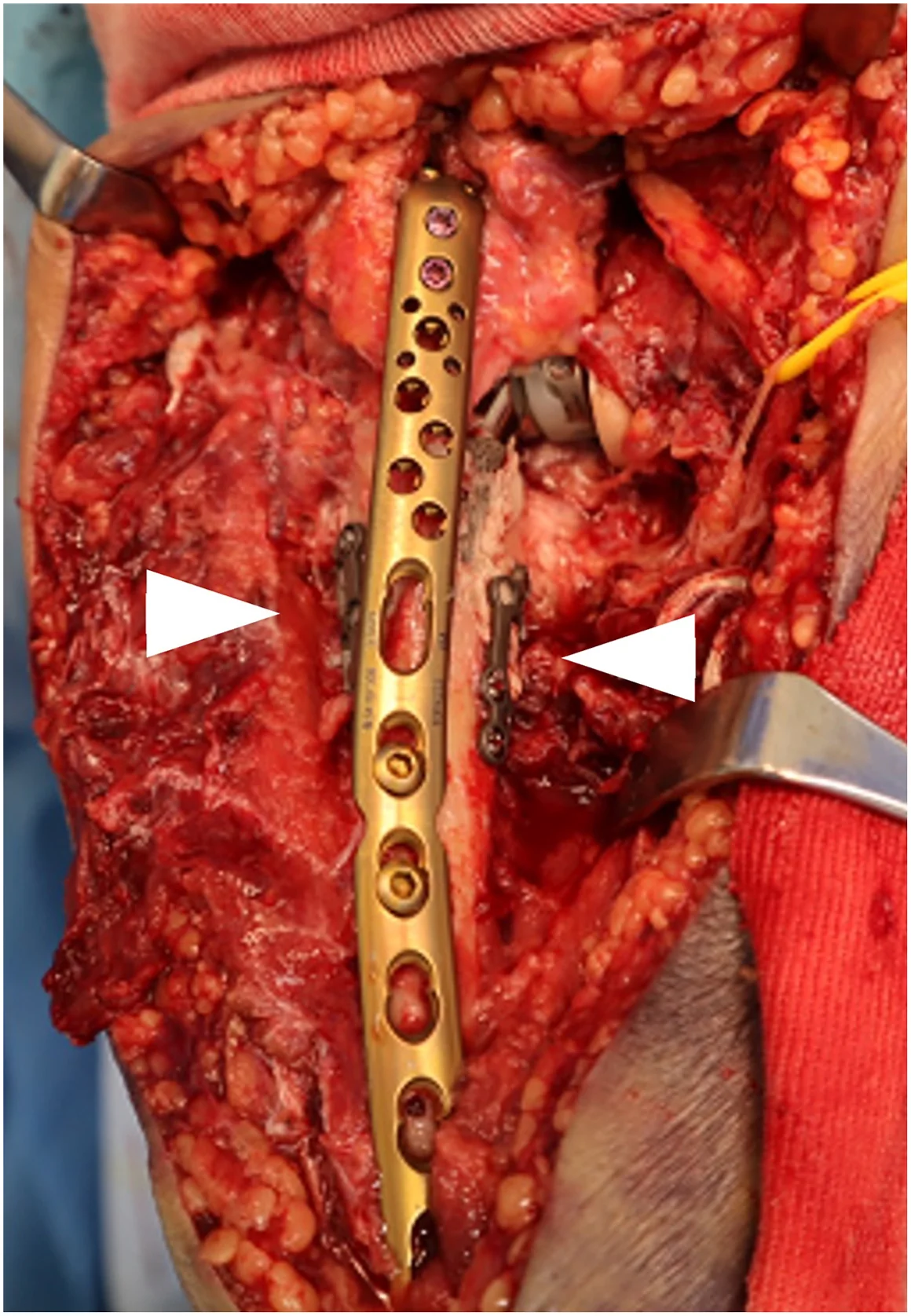
Intraoperative view of the posterior ulna showing the placement of 2 hand plates used as provisional fixation (*arrowheads*).

After thorough irrigation, the definitive humeral and ulnar components were inserted with cement. The cement was adequately pressurized during implant insertion, and any cement that overflowed from the fracture site was meticulously removed. Finally, the olecranon fragment was fixed with a locking anatomical olecranon plate (LCP Olecranon Plate 2.7/3.5, 8 holes; DePuy Synthes, West Chester, PA, USA) that was substantially longer than the ulnar component (Fig. 4). The comminuted fracture site was grafted with cancellous bone harvested from the humeral trochlea and the excised bone fragments. An intra-articular drain was placed, and the wound was closed in layers.

**Figure 4:**
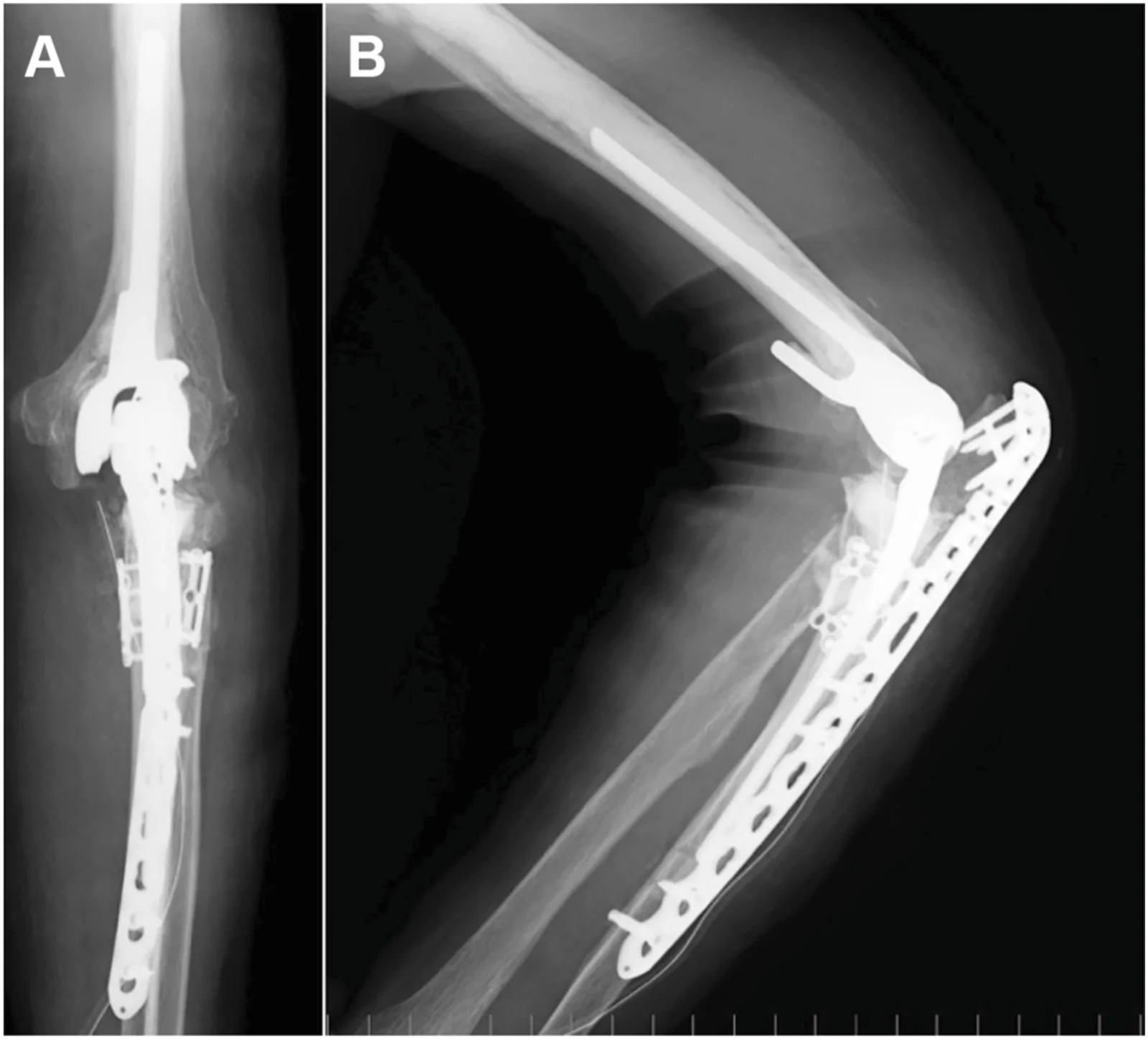
Immediately post-operative radiographs: (**A**) anterior-posterior view and (**B**) lateral view.

### Post-operative rehabilitation protocol and follow-up

Passive ROM exercises were initiated the day after surgery under the supervision of a therapist. The elbow was immobilized at 90° of flexion for 2 weeks, except during rehabilitation sessions. Subsequently, active ROM exercises were started. A lifelong lifting restriction of 2.5 kg for the operated arm was recommended. At 1 month, ROM measured −10° in extension and 120° in flexion, and the patient reported no pain (Fig. 5). Radiographic evidence of proximal ulna union was observed at 4 months. At 12 months post-operatively, imaging confirmed bone union, with no signs of prosthetic loosening or heterotopic ossification (Fig. 6). At that time, ROM was 0° extension, 135° flexion, and 90° in both pronation and supination (Fig. 7). The patient resumed her previous activity level and achieved a Mayo Elbow Performance Score (MEPS) of 100. At the final follow-up (2 years post-operatively), radiographs showed no evidence of loosening, and the clinical course remained stable (Fig. 8).

**Figure 5:**
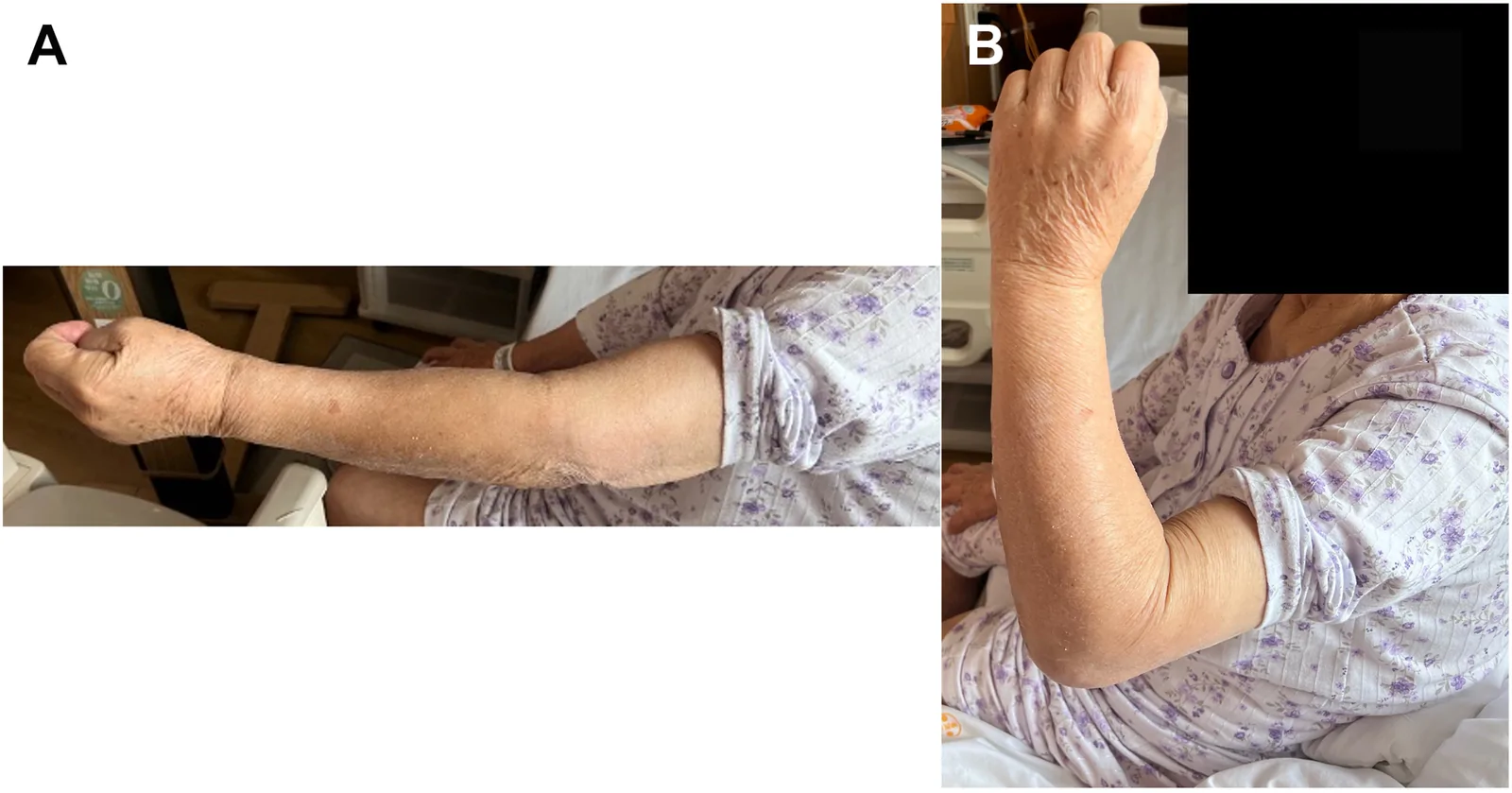
At one month post-operatively, active range of motion demonstrated −10° of extension (**A**) and 120° of flexion (**B**) with no pain.

**Figure 6:**
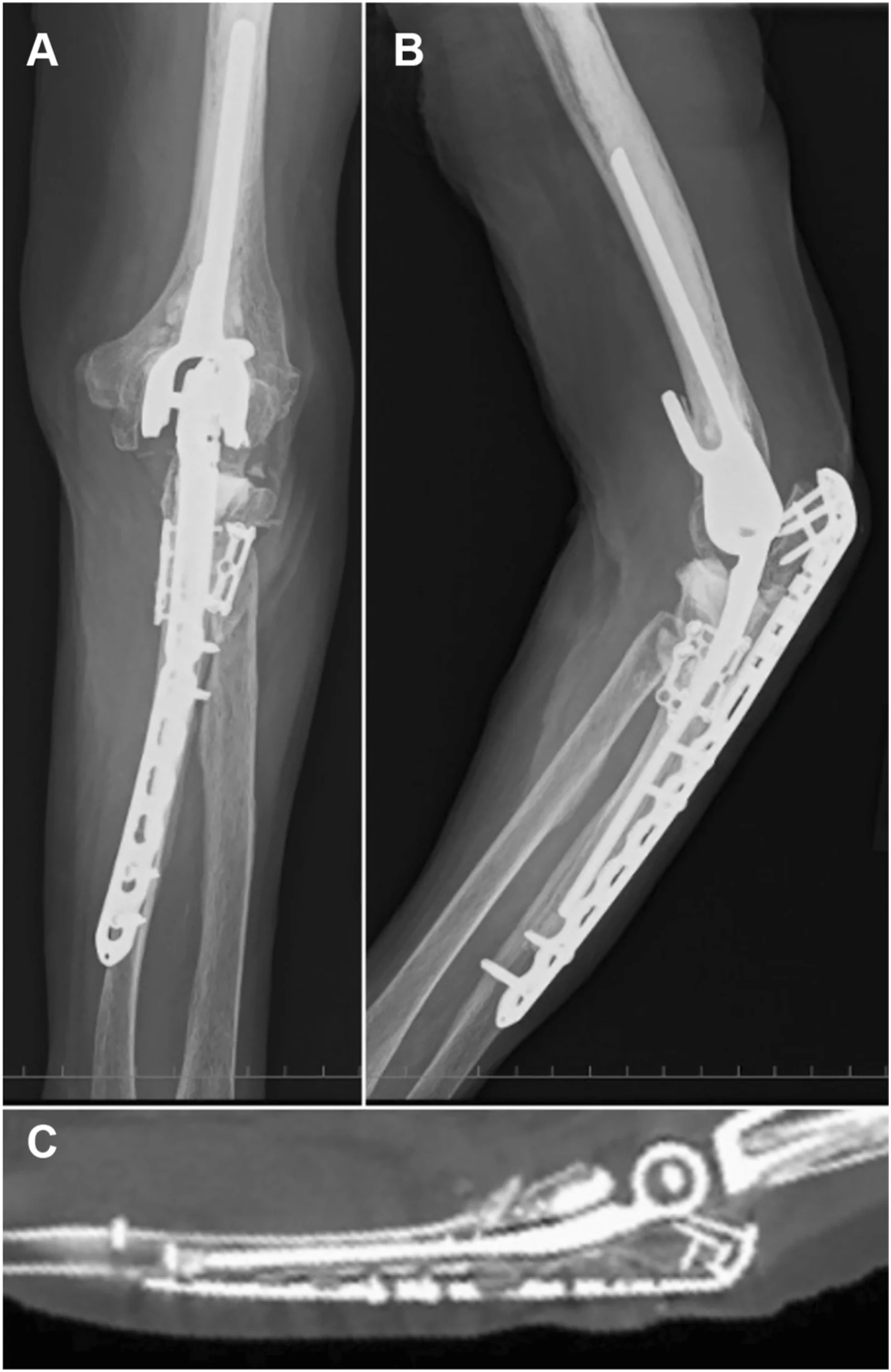
At 12 months post-operatively, radiographs showed bone union without evidence of prosthetic loosening or heterotopic ossification (**A** and **B**). Sagittal computed tomography confirmed bone union of the proximal ulna (**C**).

**Figure 7:**
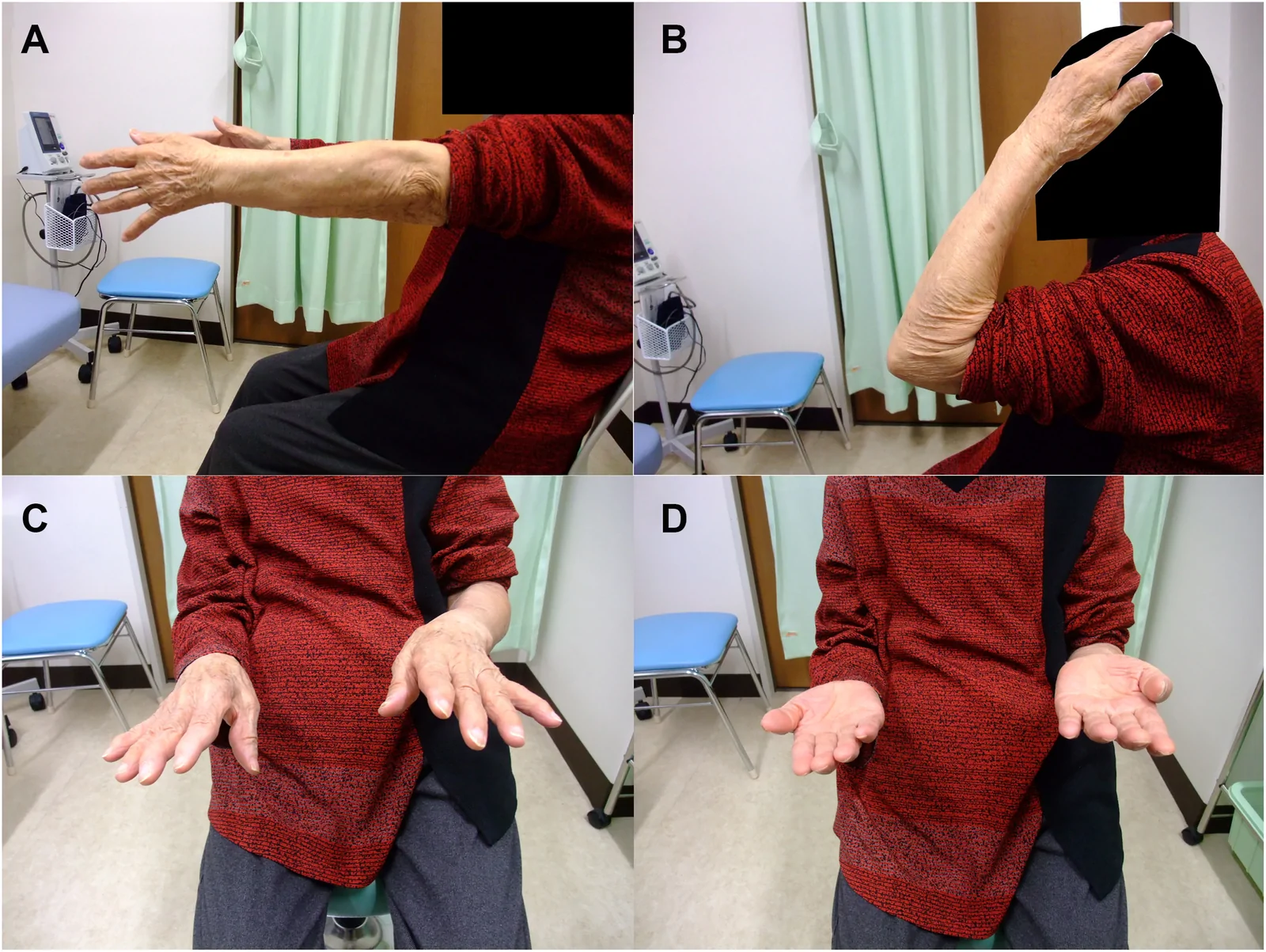
At 12 months post-operatively, active range of motion included 0° of extension (**A**), 135° of flexion (**B**), 90° of pronation (**C**), and 90° of supination (**D**).

**Figure 8:**
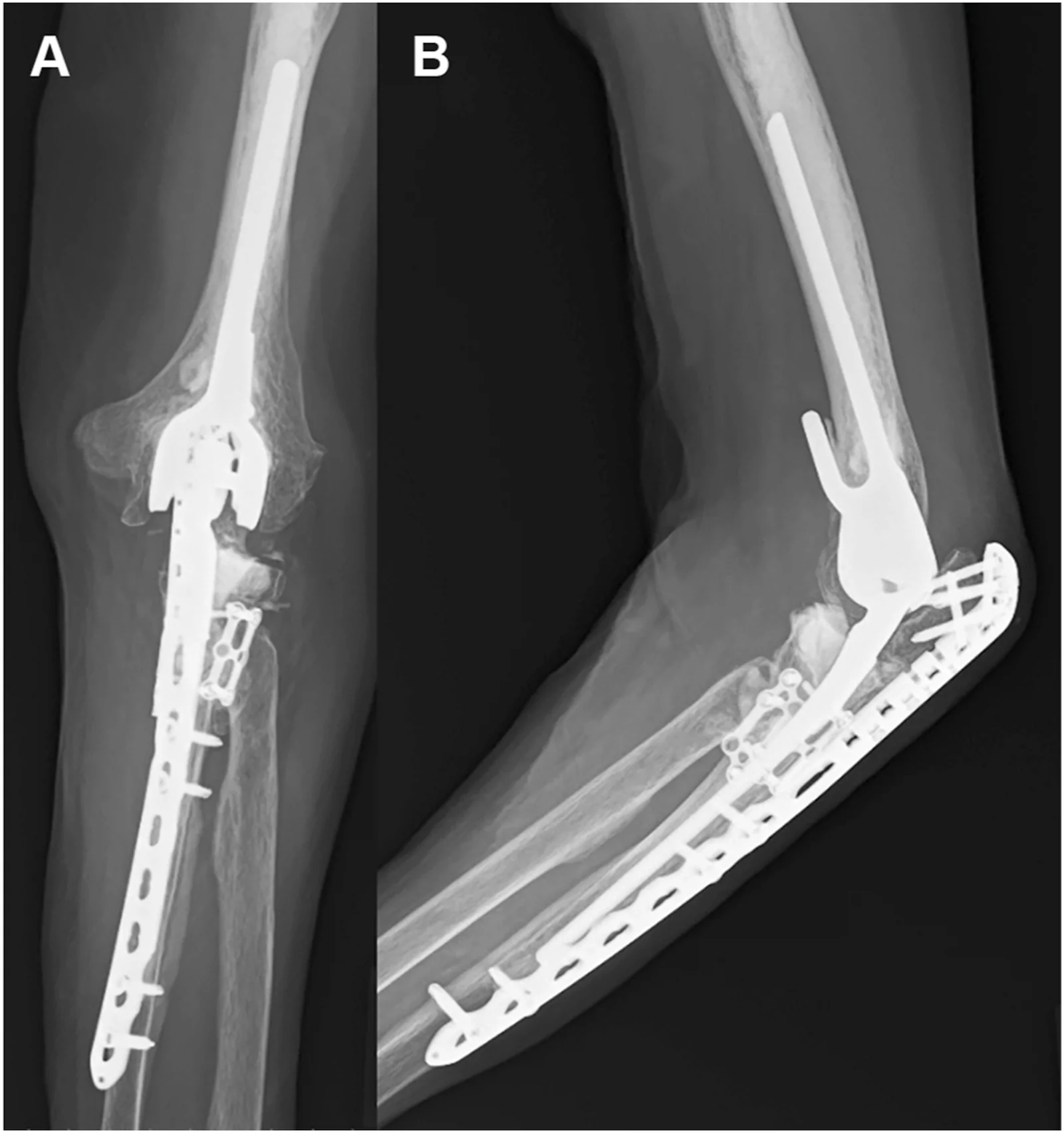
At 2 years post-operatively, radiographs showed bone union without evidence of prosthetic loosening or heterotopic ossification (**A** and **B**).

## Discussion

We treated an elderly patient with a posterior Monteggia fracture-dislocation using TEA combined with ORIF, achieving favorable outcomes. To our knowledge, there have been no previous reports describing the use of primary TEA combined with ulna fixation for a posterior Monteggia fracture-dislocation.

Current treatment protocols for type IID Monteggia fracture-dislocation follow the “fragment-specific approach” described by Beingessner et al,^3^, ^18^ in which the radial head is repaired or replaced, the ulnar diaphysis is reduced and plated, the coronoid is stabilized, the olecranon is fixed with a dorsal plate, and any ligamentous injury is addressed; a hinged or static external fixator is added if stability cannot otherwise be achieved. However, in the present case, performing ORIF was considered challenging for several reasons. First, the ulno-humeral articular fragments were too small and comminuted for anatomical reduction and stable fixation. Second, the fractures involved both the sublime tubercle, the insertion of the anterior bundle of the medial collateral ligament, and the crista supinatoris, the insertion of the lateral collateral ligament complex, making it difficult to achieve a bony reconstruction that would ensure sufficient elbow stability. Finally, severe osteoporosis further reduced the likelihood of rigid fixation, and external fixation carried an increased infection risk given the patient's poorly controlled diabetes.

To address these challenges, we performed a primary TEA combined with ORIF. A recent systematic review and meta-analysis of ORIF versus TEA for distal humerus fractures in elderly patients reported that TEA achieved better ROM and lower rates of reoperation and stiffness, with similar overall complications and function.^19^ However, reports of primary TEA for proximal forearm fractures are few, and its use for a Monteggia fracture-dislocation has not been reported. Two case reports described TEA for terrible triad injuries with excellent outcomes^16^, ^20^; our case was similar in the presence of osteoporosis but differed in the ulna fracture pattern. In our case, the fracture involved comminution of the proximal ulna, including the olecranon, coronoid, and sublime tubercle, which extended into the diaphysis. TEA alone was considered inappropriate because of the risk of ulnar component loosening and insufficient elbow extension from the olecranon fracture.^22^ We therefore stabilized the comminuted proximal ulna with plates to enhance the fixation of the ulnar component, implanted a linked-type prosthesis for stability, and applied an olecranon plate to restore elbow extension function. Similar to the olecranon osteotomy approach described previously, exposure through the olecranon fracture provided sufficient access to the joint.^23^ Bone union was successfully achieved without loosening, and the patient had a favorable clinical outcome, with a good ROM of 0° to 135° and an MEPS of 100. The patient was pain-free and experienced no complications. This outcome compares favorably with previous reports on type IID Monteggia fracture-dislocation. Guerra et al^6^ documented a mean MEPS of only 35.0 in this subtype, Konrad et al^9^ a mean Broberg-Morrey score of 67 with combined proximal ulna and coronoid fractures, and Beingessner et al^3^ preservation of a greater-than-100° arc in only 69% of patients with a 37.5% complication rate. Our case report supports TEA for complex fracture-dislocations with comminuted proximal ulna fractures including Monteggia fracture-dislocation, provided that stable fixation of the ulnar component can be ensured.

TEA for post-traumatic sequelae of proximal ulna or radius fractures and fracture-dislocations provides reliable pain relief and good function when successful; however, complication and reoperation rates are high, and the trans-ulnar basal coronoid pattern in particular is associated with the highest complication and revision rates.^24^ In a large database study of the timing of TEA for distal humerus fractures, TEA performed as a salvage procedure was associated with significantly higher rates of revision, periprosthetic fracture, and periprosthetic joint infection than primary TEA.^17^ This has been attributed to the local consequences of prior surgery and fracture healing: retained hardware, malunion or nonunion, and soft tissue scarring complicate exposure and accurate implant placement, while removal of existing hardware creates cortical defects that act as stress risers predisposing to periprosthetic fracture and loosening; a surgical field altered by previous fixation may also be more susceptible to infection.^17^ Accordingly, primary TEA may be a reasonable option for proximal forearm fracture-dislocations in carefully selected patients in whom timely anatomical reduction and stable fixation are unlikely to be achievable.

There are several disadvantages associated with the use of TEA for elbow fracture-dislocations. Its indication for fractures other than involving the distal humerus remains uncertain due to the small number of reported cases. TEA is generally not recommended for younger and/or highly active individuals, as lifelong restrictions on weight-bearing are required, and excessive use can lead to implant loosening. Complications such as bone stock loss, implant loosening, joint instability, deep infection, and periprosthetic fracture may necessitate reoperation; however, salvage options after failed TEA are limited, and outcomes are typically poor.^5^ Additionally, heterotopic ossification can still occur even after TEA.^19^, ^20^ Therefore, indications for TEA in elbow fractures should be considered with caution.

A limitation of this case is that the follow-up period was relatively short, and long-term outcomes remain unknown. Further studies with extended follow-up are needed to better understand the efficacy and potential role of TEA in treating elbow fractures beyond those of the distal humerus.

## Conclusion

Primary TEA combined with ORIF achieved a favorable clinical outcome in an elderly patient with a posterior Monteggia fracture-dislocation. To our knowledge, this is the first reported case of primary TEA for this injury pattern. Primary TEA combined with ORIF may be a valuable treatment option for elderly patients in whom anatomical reconstruction and stable fixation are unlikely to be achieved with ORIF and ligament repair alone.
